# Sildenafil-induced priapism in a dog : an unusual case report

**DOI:** 10.1186/s12917-024-04205-6

**Published:** 2024-08-08

**Authors:** Hyo-Seung Nam, Ye-In Oh

**Affiliations:** 1 Dasom Animal Medical Center, 3, Suyeong-ro 13 Beon-gil, Nam-gu, Busan, Republic of Korea; 2 Department of Veterinary Internal Medicine, College of Veterinary Medicine and Institute for Veterinary Biomedical Science, Kyungpook National University, Daehakro 80, Daegu, 41566 Republic of Korea

**Keywords:** Dog, Sildenafil, Priapism, Pulmonary hypertension

## Abstract

**Background:**

Priapism is defined as erection that lasts for more than 4 h without sexual stimulation. There are various causes of priapism, but there are no reports of sildenafil-induced priapism in dogs. In human medicine, there were no pre-marketing reports of priapism caused by sildenafil, but post-marketing surveillance has shown that it is rare. In cases of pulmonary hypertension in dogs, sildenafil is the first-line drug of choice for symptomatic relief.

**Case presentation:**

An 11-year-old neutered male Maltese dog that presented with tachypnea and cough was diagnosed with myxomatous mitral valve disease, American College of Veterinary Internal Medicine (ACVIM) stage C, and was treated medically. Eighteen months after the diagnosis, severe pulmonary hypertension occurred due to left heart disease. At 20 months postdiagnosis, pleural effusion occurred, and sildenafil (2 mg/kg twice daily) was added to the existing treatment. Two weeks later, the dyspnea recurred, confirming pleural fluid recurrence, and sildenafil was increased to 2 mg/kg thrice daily. One day later, the patient developed persistent erections and penile pain. Penile amputation and urethrostomy were recommended but were refused; therefore, analgesia and palliative care were provided. The patient died of acute dyspnea 22 months after the first presentation, with no specific priapism recurrence at the time of death.

**Conclusion:**

To the best of our knowledge, this is the first report of sildenafil-induced priapism in a dog with pulmonary hypertension.

## Background

Priapism is an erection maintained for more than four hours that occurs without sexual stimulation [1]. Priapism in dogs is rare and has been reported for reasons related to T12–T13 hemilaminectomies and idiopathic acute disc extrusion, secondary to lumbar meningomyelocele or lumbar stenosis [2–4].

Although the etiology of priapism in humans is variable, reports of sildenafil-induced priapism are rare in post-marketing reports [5–7]. Sildenafil, a phosphodiesterase type 5 inhibitor, blocks the breakdown of cGMP, increasing its levels and causing an erection by relaxing the smooth muscles in the corpus cavernosum, drawing in blood, and compressing peripheral veins. Because of these pharmacological benefits, it is primarily used in humans for erectile dysfunction [8]. However, due to their role as vasoactive erectile agents, they can also cause priapism [7]. Additionally, phosphodiesterase type 5 is more abundant in the pulmonary vasculature, preferentially dilating pulmonary vessels [9]. Sildenafil has been used to alleviate clinical symptoms of canine pulmonary hypertension, but its use in pulmonary hypertension due to left heart disease is avoided as it may worsen left-sided heart failure. However, if clinical manifestations of pulmonary hypertension, such as pleural effusion or syncope, are evident, sildenafil may be considered [10]. In the present case, sildenafil was administered for the same reason, and priapism occurred after administering sildenafil (2 mg/kg three times a day [tid]). To date, priapism due to sildenafil use in dogs has not been reported; to our knowledge, this is the first report of this condition. Although sildenafil is a commonly used medication without serious side effects, we should be aware of the following rare side effects, which may also occur.

## Case description

An 11-year-old neutered male Maltese dog weighing 3 kg presented with tachypnea and cough as primary symptoms and was diagnosed with myxomatous mitral valve disease with mitral valve chordae tendineae rupture (American College of Veterinary Internal Medicine [ACVIM] stage C). The patient had been consistently taking preventive measures against heartworm and had not received the core vaccines. The patient was prescribed pimobendan (0.25 mg/kg twice daily [bid]), furosemide (1.5 mg/kg bid), spironolactone (1 mg/kg bid), and enalapril (0.5 mg/kg bid). This treatment resolved his clinical symptoms [11, 12].

**Fig. 1:**
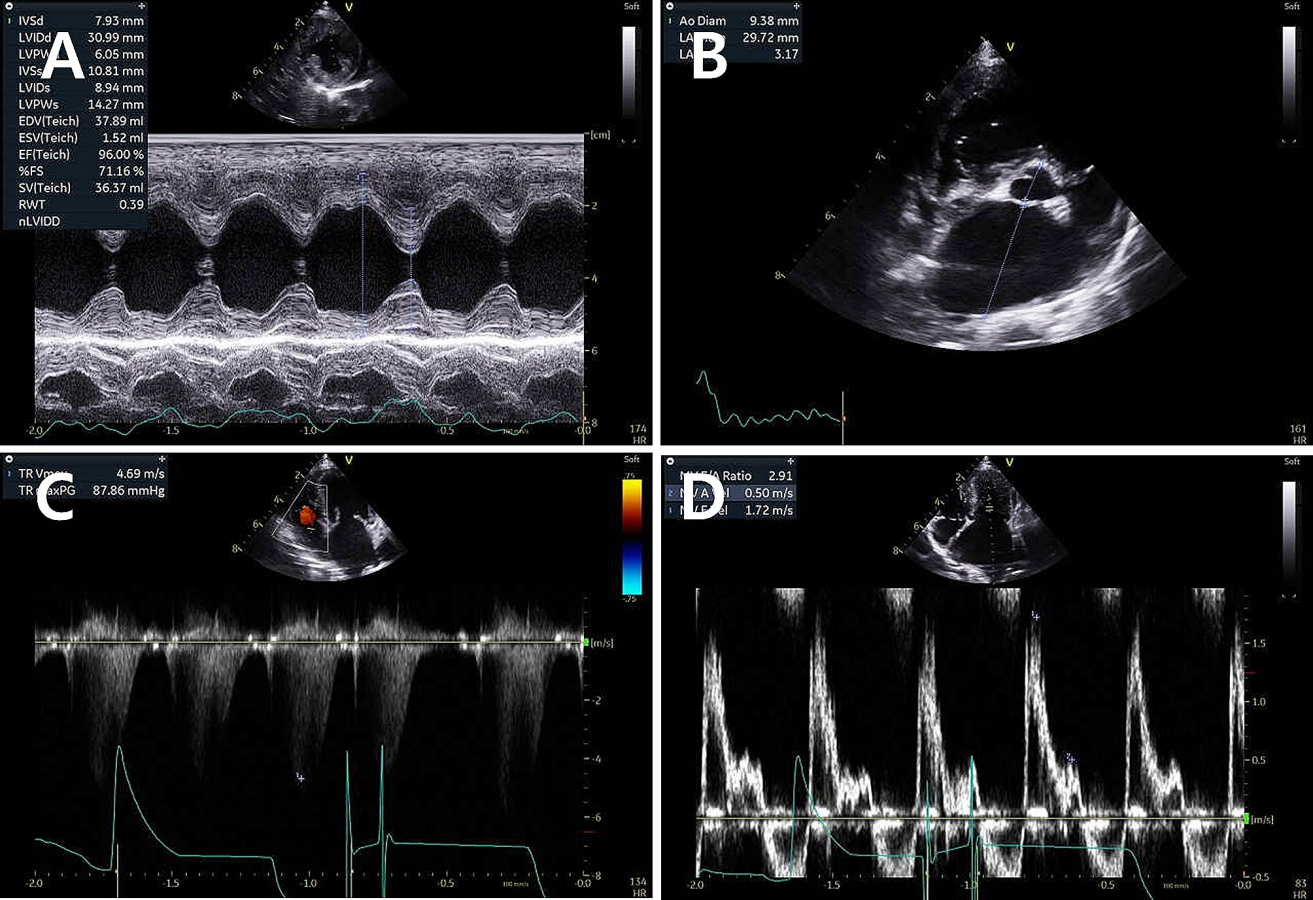
Echocardiography at 18 months after the first presentation. (**A**) The normalized left ventricular internal diameter during diastole (LVIDDN) is 2.18 from the right parasternal short-axis view. (**B**) 2D left atrial–aortic ratio (LA: AO) is 3.17 from the right parasternal short axis view. (**C**) Tricuspid valve regurgitation velocity is 4.69 m/s from the left apical four-chamber view. (**D**) Transmitral valve E velocity is 1.72 m/s from the left apical four-chamber view

**Fig. 2:**
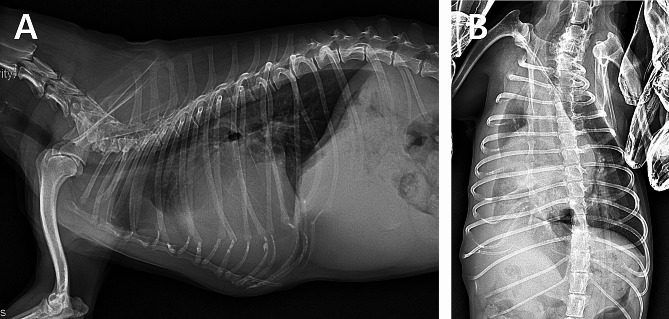
Chest radiographs of the dog showing pleural effusion. (**A**) Right lateral chest radiograph depicting the scallop sign. (**B**) Ventrodorsal view of chest depicting a pleural effusion in the right pleural cavity

At 18 months following the first presentation, the sleeping respiratory rate of the patient was stable, and there was no cough. However, the dog developed clinical symptoms related to syncope and visited our clinic. Echocardiography revealed an enlarged left ventricular internal diameter in diastole, normalized for body weight (LVIDDN 2.18), two-dimensional left atrial-aortic ratio (LA: AO 3.17), and elevated transmitral valve E velocity (Epeak 1.72 m/s), with a peak tricuspid regurgitation velocity of 4.69 m/s; hence, indicating severe pulmonary hypertension due to left heart disease (Fig. 1) [10–12]. Despite syncope being the primary symptom, these elevated measurements suggested that sildenafil could have worsened the left-sided heart failure. Consequently, the patient was treated with furosemide (2.5 mg/kg) and pimobendan (0.35 mg/kg bid). Twenty months after diagnosis, the patient returned with worsening syncope and dyspnea. A chest radiograph revealed pleural effusion in the right thoracic cavity, leading to thoracocentesis and the removal of 150 mL of pleural effusion (Fig. 2). Sildenafil (2 mg/kg bid) was added to the existing medication. The patient was subsequently free of syncope but returned 2 weeks after the thoracocentesis with recurrent dyspnea. Thoracocentesis was performed again, resulting in the removal of 230 mL of pleural fluid, and the sildenafil dose was increased to 2 mg/kg tid.

**Fig. 3:**
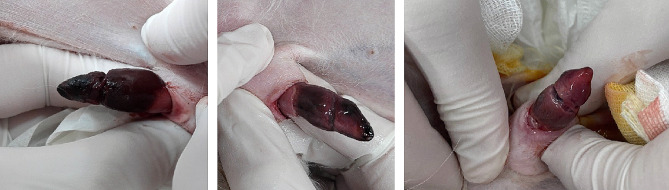
Penile lesion of the dog with severe necrosis and pain

One day after escalation to sildenafil 2 mg/kg tid, the patient developed a persistent erection and severe penile pain. Physical examination revealed that both the glans penis and bulb of the glans penis were already necrotic, with only the ventral side of the penis having some normal color (Fig. 3). At that time, the patient’s rectal temperature was 38.3 °C, respiratory rate was 48 breaths per minute, and heart rate was 150 beats per minute.

Penile amputation and urethrostomy were recommended. However, due to the severity of the patient’s underlying cardiac disease and refusal of surgical treatment, analgesia and supportive care were provided. The area around the penis was disinfected with 1 mL of povidone-iodine diluted in 19 mL of sterile 0.9% normal saline. Carprofen 2.2 mg/kg bid and gabapentin 10 mg/kg bid were prescribed for analgesia and inflammation, and amoxicillin-clavulanic acid 12.5 mg/kg bid was prescribed as a prophylactic antibiotic. Sildenafil, which was thought to be the cause of priapism, was discontinued.

**Fig. 4:**
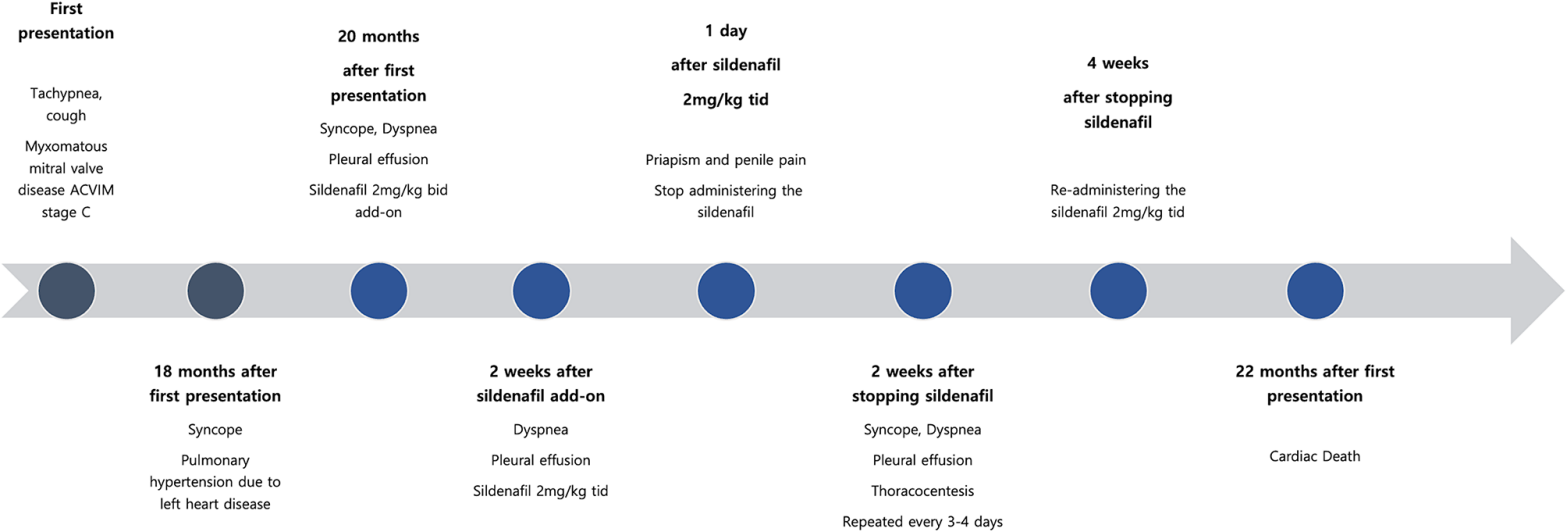
Patient timeline. Eighteen months after being diagnosed with myxomatous mitral valve disease ACVIM stage C, pulmonary hypertension due to left heart disease developed. Sildenafil was administered at the onset of dyspnea due to pleural effusion, and ischemic type priapism occurred when sildenafil was given at a dose of 2 mg/kg tid

Two weeks after the presentation with priapism, the necrotic tissue auto-amputated, exposing part of the os penis. No further pain, penile necrosis, or urination problems were observed. Two weeks after sildenafil discontinuation, the clinical symptoms of dyspnea and fainting recurred. Therefore, 250–300 mL of pleural effusion was removed via thoracocentesis every 3–4 days. Considering the patient’s quality of life, sildenafil 2 mg/kg tid was restarted one month after sildenafil discontinuation. There were no subsequent recurrences of priapism, and sildenafil was titrated according to the clinical presentation. The patient died of acute dyspnea 22 months after the initial presentation; priapism did not recur (Fig. 4).

## Discussion and conclusion

Priapism, which refers to a persistent erection, can be divided into three main categories: ischemic (low-flow), nonischemic (high-flow), and stuttering priapism, which is recurrent priapism [1, 2]. Stuttering priapism falls within the category of ischemic type and is defined as the periodic recurrence of an erection within 3 h [4]. In human medicine, the causes of the ischemic type include sickle cell disease and other hemoglobinopathies, vasoactive drugs, hematological dyscrasias, neoplastic diseases, hemodialysis, hyperlipidemic parenteral nutrition, heparin treatment, Fabry’s disease, and neurological diseases (spinal cord damage, and anesthesia). Nonischemic priapism can be caused by trauma, vasoactive drugs, penile revascularization surgery, or neurological conditions [1]. In this study, the cause (vasoactive drugs) led to progression and necrosis on visual assessment; Hence, despite not collecting blood from the corpus cavernosum for blood gas analysis, it was evident that the condition represented an ischemic (low-flow) priapism.

Evaluation of cavernous blood gas can help distinguish between ischemic type and nonischemic type. Ischemic types will show a pH < 7.25, aPO_2_ < 30 mmHg, and PCO_2_ > 60 mmHg, whereas nonischemic types will typically show a pH of 7.4, aPO_2_ > 90 mmHg, and aPCO_2_ < 40 mmHg [1, 13]. Metabolic factors, including hypoxia, cause irreversible damage to the smooth muscle, eventually leading to erectile tissue dysfunction. [1] Therefore, the ischemic priapism represents an emergency that requires treatment as soon as possible [4]. Conservative treatment may include aspiration of blood stagnant in the corpora cavernosa and injection of an adrenergic agent directly into the corpora cavernosa [2]. In this case, penile necrosis was visibly advanced and required penile amputation and urethrostomy. However, due to the severity of the patient’s medical situation and the declining of surgical treatment, analgesia and palliative care were provided. Sildenafil, which was thought to be the cause of priapism, was discontinued; however, the treatment had to be restarted one month after discontinuation. Priapism did not recur upon restarting sildenafil. In humans, erectile dysfunction is known to occur if ischemic priapism is not corrected quickly [7]. It is believed that priapism did not recur because of the previous injury. Generally, sildenafil is regarded as a medication that can be administered with minimal side effects [8]. In humans, it is primarily used for erectile dysfunction. Apart from this, it is most commonly used for pulmonary hypertension. In cases of pulmonary hypertension due to left heart disease, sildenafil is avoided because of its potential to worsen left-sided heart failure. However, if a patient presents with clinical symptoms associated with overt pulmonary hypertension, such as pleural effusions, ascites, or syncope, sildenafil may be considered with careful monitoring for left-sided heart failure [10]. In this case, after delaying the use of sildenafil for as long as possible, the patient presented with severe syncope and pleural effusions. Sildenafil was administered, and clinical improvements were observed. During the subsequent dose escalation process, clinical symptoms related to priapism were identified after 1 day of administering sildenafil (2 mg/kg tid), and physical examination revealed that severe penile necrosis had already developed. Decompression has been attempted to treat ischemic priapism in humans but was not performed in this case.

Aspiration is carried out to decompress the corpora cavernosa, continuing until no more dark but fresh blood is extracted. This process significantly reduces intracavernous pressures, relieves pain, and facilitates recovery. Following the procedure, an adrenergic agent is infused intracavernously. Available medications include ephedrine, epinephrine, etilefrine, metaraminol, and phenylephrine. Shunt surgery can be considered [7]. In veterinary medicine, penile amputation and urethrostomy are performed on patients with stuttering priapism [2]. There is also evidence of oral pseudoephedrine use in cases of non-ischemic priapism [4]. However, there is a lack of evidence regarding treatment options in veterinary medicine.

To the best of our knowledge, this is the first report of priapism following sildenafil administration in dogs. At presentation, the dog had severe penile necrosis and pain; thus, suggesting ischemic priapism. Sildenafil is a drug that can still be reliably used to relieve clinical symptoms in canine pulmonary hypertension. However, ischemic priapism can occur in dogs following sildenafil administration; therefore, its use requires careful monitoring.

## Data Availability

No datasets were generated or analysed during the current study.

## Abbreviations

ACVIM: American College of Veterinary Internal Medicine
bid: Twice daily
cGMP: Cyclic Guanosine Monophosphate
Epeak: Transmitral valve E velocity
LA:AO: Left Atrial-Aortic Ratio
LVIDDN: Left Ventricular Internal Diameter in Diastole, Normalized for body weight
tid: Three times a day
